# Observations on Conception and Pregnancy in the Mare1Read before the Michigan State Veterinary Medical Association, February 5, 1896.

**Published:** 1897-03

**Authors:** J. A. Dell

**Affiliations:** Ann Arbor, Michigan


// THE JOURNAL

OF

COMPARATIVE MEDICINE AND VETERINARY ARCHIVES.

VOL. XVIII. *MARCH*, 1897. No. 3.

### SOME OBSERVATIONS ON CONCEPTION AND PREGNANCY IN THE MARE.[1]

BY J. A. DELL, V.S.,
ANN ARBOR, MICHIGAN.

CONSIDERING the number of patrons and the great number of patients that the veterinarian is called to serve and attend, but a small percentage of consultations can be classed as pertaining to the reproduction of the species he is called to treat. We are called to treat sterility and the diseases and accidents of pregnancy, more particularly those of the later stages. Occasionally a patron calls our attention to a mare supposed to be pregnant, and asks: "What do you think? Is she in foal?" It is seldom he expects to pay for the opinion and more seldom that the practitioner expects he will. This may be one reason why he is so often answered "I think so and so."

On behalf of the farmer, I think he may be pardoned for asking some of us what we think, for the simple reason so many veterinarians are non-committal on this subject, or admit that there is nothing definite until, to use the stable term, "you see the colt kick," which is not much before the eighth or ninth month of pregnancy.

The farmer or breeder who has grown up in the country has certain traditional rules or signs by which he is guided, but these rules are not always reliable. For instance, he tests his mare until bred, or breeds her on or about the ninth day after foaling; tests her in three weeks, and, if she refuses, every week for four or six weeks. If she refuses the stallion through three periods, she is believed to be in foal and is treated likewise. In the meantime

[1] Read before the Michigan State Veterinary Medical Association, February 5, 1896.

it is noticed if she is dull and tends to flesh; if the crest of her neck gets more firm; if cross to other horses; if the abdomen enlarges; and, later, if the udder swells. Still nothing definite is known until the ninth or tenth month, when the movements of the fœtus become so active that they are easily seen. But other movements of the walls of the abdomen have been mistaken for those of the fœtus, and after observing these symptoms the attendants of mares more or less valuable have faithfully watched weeks and months but no foal appeared.

The relating of such failures to me by breeders, together with complaints against owners of stallions that stood by the season, that they did not give due attention to mares left in their charge, have led me to record some observations on the time of conception and the signs of pregnancy, to ascertain the earliest period when I could give a definite opinion if a mare was or was not pregnant. These observations have extended over several seasons, and include mares of all ages and sizes, from draft to a Shetland pony, all in good health for breeding.

To ascertain the length of the term of œstrum, or heat, I caused mares to be tested each day until it appeared, then tested or bred them each day as long as they would receive the stallion; and in forty cases so tested the shortest was one day, the longest eighteen days. I might add that in the latter case conception took place, while in the former it did not. Excepting this single instance, all continued for four days or over—a few over ten days; but they averaged about eight days, and I believe that can safely be given as the average for mares kept in this climate.

By breeding each day, and testing and not breeding, I find that copulation does not have any tendency to shorten the period. This, to my mind, should dispel the idea entertained by most writers that the heat subsides when conception takes place. Furthermore, I believe that the same number of ova are developed during the period whether copulation takes place or not, just as oviparous animals produce the same number of eggs whether the male fertilizes them or not. Again, many of us know of instances where a bitch had been lined, and several days thereafter had broken away and been lined by a dog of a different breed, and when the pups were born two kinds were in the litter. I have bred mares at the very beginning of the heat; tested, I found them to continue in heat their usual time, yet became pregnant. But in my observations only a small percentage became pregnant if they continued in heat four days or over, and this leads me to believe that the ordinary life of the free spermatozoa is not much beyond three or four days; and mares bred and showing signs of heat on the fourth day, in my opinion, should be bred again to insure pregnancy.

The number of days elapsing between the beginning of one heat and the appearance of the next, like the number of days it continues, varies much. My records show the shortest to be thirteen and the longest twenty-eight days, while a few vary slightly, such as thirteen, fifteen, nineteen to twenty-one, twenty-six to twenty-eight days. It comes so closely to some multiple of seven, that I think we can safely look for its reappearance in fourteen, twenty-one, or twenty-eight days.

I find that these periods are peculiar to the same mare; that is, if it appears in a mare in fourteen days we can expect it at these intervals; but by far the majority can be rated at twenty-one days. I think a fair estimate would be 75 per cent. at twenty-one, 20 per cent. at fourteen, and 5 per cent. at twenty-eight days.

In mares that have foals we are again somewhat at sea as to the number of days that intervene between foaling and the first period of œstrum. Beginning the test as early as the fourth day I have never seen it earlier than the seventh day, and the latest it has appeared during the suckling period was the forty-sixth day. Some mares—but if watched closely, there are very few—show no signs of heat after birth until the foal is weaned.

In the majority of mares it appears on or about the ninth day; but still there are so many a few days earlier or later that it may be looked for any time from the seventh to the twenty-eighth day. Very few go beyond the latter; and as the period of its continuance is liable to be much shorter than the subsequent heats, I believe we should advise testing at least twice a week during that period.

Although in cold weather, in stables not artificially heated, the reappearance of the heat may be deferred even to the passing of one or more periods, in weather such as we have in Michigan from May 1st to October 1st, I have never met with an instance where a mare bred every day or every third day during the heat, and tested as often as twice a week thereafter, and refusing up to and beyond her usual time for its reappearance, did not prove to be pregnant. But to avoid failure from a peculiarity of certain mares I would advise testing twice a week for six to eight weeks; that would take them beyond the second period.

To illustrate the regular reappearance of heat, the length of time it may last, and the results that may be reached by persistent breeding, I will relate one instance: On April 2, 1895, a mare of mine foaled a colt. I did not get the stallion to which I wished to breed her until April 27th. On that date she proved to be in heat; I also believe she was in heat about the twelfth day after foaling, but did not test her. She was bred on the 27th, and each day until May 5th, making nine days. On the 6th she refused, and refused every day until May 11th, fourteen days after first service. She was bred every day until May 17th, seven days. She came in heat again on May 24th, thirteen days after beginning of second heat, and was bred each day until May 30th, seven days, and after refusing each day for fourteen days was then tested twice per week for six weeks, I was able to detect life in fœtus October 30th, five months after last service.

(To be continued.)

